# An integrated n-Si/BiVO_4_ photoelectrode with an interfacial bi-layer for unassisted solar water splitting†

**DOI:** 10.1039/d2sc06651c

**Published:** 2023-01-30

**Authors:** Shujie Wang, Shijia Feng, Bin Liu, Zichen Gong, Tuo Wang, Jinlong Gong

**Affiliations:** a School of Chemical Engineering and Technology, Key Laboratory for Green Chemical Technology of Ministry of Education, Tianjin University Tianjin 300072 China jlgong@tju.edu.cn wangtuo@tju.edu.cn; b Collaborative Innovation Center of Chemical Science and Engineering (Tianjin) Tianjin 300072 China; c Haihe Laboratory of Sustainable Chemical Transformations Tianjin 300192 China; d Joint School of National University of Singapore and Tianjin University International Campus of Tianjin University, Binhai New City Fuzhou 350207 China; e National Industry-Education Platform of Energy Storage Tianjin 300350 China

## Abstract

Integrated n-Si/BiVO_4_ is one of the most promising candidates for unbiased photoelectrochemical water splitting. However, a direct connection between n-Si and BiVO_4_ will not attain overall water splitting due to the small band offset as well as the interfacial defects at the n-Si/BiVO_4_ interface that severely impede carrier separation and transport, limiting the photovoltage generation. This paper describes the design and fabrication of an integrated n-Si/BiVO_4_ device with enhanced photovoltage extracted from the interfacial bi-layer for unassisted water splitting. An Al_2_O_3_/indium tin oxide (ITO) interfacial bi-layer was inserted at the n-Si/BiVO_4_ interface, which promotes the interfacial carrier transport by enlarging the band offset while healing interfacial defects. When coupled to a separate cathode for hydrogen evolution, spontaneous water splitting could be realized with this n-Si/Al_2_O_3_/ITO/BiVO_4_ tandem anode, with an average solar-to-hydrogen (STH) efficiency of 0.62% for over 1000 hours.

## Abstract 2

This paper describes an interfacial bi-layer for the integration of Si and BiVO_4_, predicted to achieve record total H_2_ production for unbiased solar water splitting.

## Graphical abstract

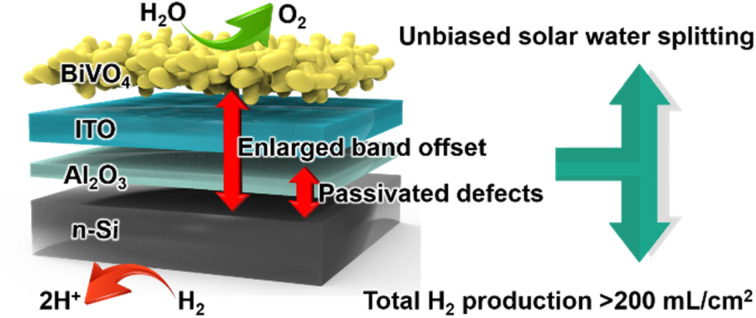

## Introduction

Water photolysis is a potential carbon-neutral sustainable energy technology to attain solar to chemical energy conversion.^1–4^ Unbiased water splitting powered only by solar energy is of great significance for the effective use of solar energy, which includes photoelectrochemical (PEC) and photovoltaic-electrolysis (PV-EC) systems.^5,6^ Despite the higher solar-to-hydrogen (STH) efficiency and stability of PV-EC systems compared with current PEC systems, due to the maturity of PV technology, additional AC/DC microgrids are needed in a PV-EC system that may reduce its economic competitiveness.^7^ PEC devices present a higher degree of system integration, in which photo-absorbers and catalysts could be integrated as a monolithic device, but it imposes several key criteria on the photoelectrode:^8–10^ (1) an appropriate bandgap to effectively utilize the solar spectrum; (2) sufficient photovoltage to drive water redox reactions; (3) high-quality contacts for carrier separation and transfer. However, there has been no ideal single photoelectrode material that satisfies all these criteria to date. Theoretical STH efficiency calculations indicate that a more promising approach is to employ a dual-absorber tandem cell with band gaps of ≈1.2 and 1.8 eV.^11^ For small band gap semiconductors (CIGS,^12^ Cu_2_O,^13^ Sb_2_Se_3_,^14^*etc.*), crystalline silicon (Si) is an excellent choice due to its narrow bandgap (1.12 eV), earth abundance, and mature production technology.^15,16^ Among the wide band gap semiconductors (WO_3_,^17^ TiO_2_,^18^ Fe_2_O_3_,^19^*etc.*), BiVO_4_ has attracted much attention for pairing with Si, motivated by its suitable band gap (≈2.4 eV), low cost, and large photovoltage (>1 V) generated from the semiconductor–liquid junction.^20,21^ There are three configurations for the series connection of Si and BiVO_4_, including photovoltaic–photoanode (PV/PEC),^22^ photocathode–photoanode (PEC/PEC)^23^ and integrated PEC cell (Integ. PEC).^24^ The Integ. PEC cell combines two semiconductors into a wireless photoelectrode, which improves flexibility in device design, giving it the potential to scale up.^6,25,26^ Unfortunately, only a few reported integrated Si/BiVO_4_ PEC tandem cells, where BiVO_4_ was grown directly on top of Si, have achieved unbiased water splitting with solar-to-hydrogen (STH) efficiencies below 0.5%.^24,27^

The overall energy conversion efficiency of integrated unbiased water splitting is largely restricted by the sluggish carrier transport at the Si/BiVO_4_ interface. Due to the small carrier diffusion length of BiVO_4_ (∼70 nm), nanoporous BiVO_4_ with an average particle size of ∼100 nm is usually fabricated.^28^ However, the porous nature of BiVO_4_ cannot prevent the bottom Si layer from being oxidized to form an insulating SiO_*x*_ interfacial layer,^29^ which increases the resistance of carrier transport, hindering the carrier transport between Si and BiVO_4_. Moreover, a considerable number of carriers will be trapped in the defects at the Si/SiO_*x*_/BiVO_4_ interfaces, leading to significant interfacial recombination that deteriorates carrier transport.

A promising approach to address the carrier transport bottlenecks at the Si/BiVO_4_ interface is to introduce an interfacial layer to connect the individual absorbers.^30^ Compared to the performance of BiVO_4_ grown on conductive F-doped SnO_2_ (FTO) glass (FTO/BiVO_4_ photoanode), the n-Si/BiVO_4_ tandem photoanode with a TiO_2_ (ref. 29) and SnO_2_ (ref. 31) interfacial layer allows a cathodic shift of onset potential by 0.3 and 0.35 V, respectively, where the TiO_2_ or SnO_2_ layer plays multiple roles in (1) preventing the detrimental oxidation of Si, (2) healing the defects at the Si/BiVO_4_ interface, and (3) constructing a heterojunction with n-Si to increase the interfacial barrier height. To satisfy the roles, the interfacial layer should be thick enough, for example, 20–25 nm for the n-Si/TiO_2_/BiVO_4_ photoanode; however, this increases the carrier transport resistance due to the relatively poor electrical conductivity of TiO_2_.^29^ Thus, there is a trade-off between the suppression of interfacial recombination and effective transport of carriers. Moreover, the photovoltage generated by the insertion of TiO_2_ and SnO_2_ layers is still insufficient to drive the overall water splitting.^29,31^

A buried p–n homojunction is another effective route to improve the photovoltage by forming a doped layer.^32,33^ It has been demonstrated that tandem photoanodes consisting of a core–shell structured np^+^-Si/BiVO_4_ nanowire array^24^ or np^+^-Si/WO_3_ microwire array^34^ were capable of unbiased solar water splitting, in which the np^+^-Si contributed more than 0.5 V to the photovoltage. However, nanostructured Si with a high surface area may also induce high surface recombination,^35^ which requires a discrete intermediate third material (SnO_2_, indium tin oxide (ITO), *etc.*) to passivate the defects. In addition, the practical application of nanostructured np^+^-Si may be hampered by the relatively complex photolithography-based pattern transfer techniques.^36,37^

Apart from p–n homojunctions, Si-based metal–insulator–semiconductor (MIS) junctions have triggered a lot of interest owing to their facile fabrication and the potential to achieve a larger band offset between the metal and semiconductor.^38,39^ The barrier height of an n-Si based MIS junction is determined by the difference between the work function (*Φ*_m_) of the metal layer and the electron affinity (*χ*_s_) of n-Si (4.05 eV).^3^ Inserting a high work-function Pt (*Φ*_m_ 5.65 eV^40^) layer in an n-Si/WO_3_ heterojunction would elegantly create a new n-Si/Pt Schottky junction that effectively increases the band bending of n-Si for photovoltage generation.^41^ Unfortunately, Pt may also form a Schottky barrier with WO_3_, which impedes the flow of electrons from WO_3_ to Pt.^41^ Moreover, the optical loss caused by the Pt layer is also an inevitable problem. Therefore, it is crucial to find a method to employ a transparent interfacial layer with an appropriate work function to develop a large Schottky barrier with the n-Si side for photovoltage extraction, while preventing the formation of a Schottky barrier with the BiVO_4_ side for effective carrier transport, which may fully utilize Si and BiVO_4_ for unassisted overall solar water splitting.

This paper describes the design and fabrication of an interfacial bi-layer for an n-Si/BiVO_4_ integrated device to achieve unassisted water splitting. Specifically, an Al_2_O_3_/ITO bi-layer was inserted between n-Si and BiVO_4_ to promote carrier transport, where the metallic ITO acted as a high-work function layer to enlarge the interfacial band offset with n-Si, as well as forming an ohmic contact with BiVO_4_, while the Al_2_O_3_ layer acted as a passivation layer to eliminate the interfacial defects between n-Si and ITO. Upon the adoption of the interfacial bi-layer, an n-Si/Al_2_O_3_/ITO MIS junction was formed, which enhanced the photovoltage of the tandem cell by 0.53 V compared with ITO/BiVO_4_. Thus, this n-Si based photoelectrode could be connected with BiVO_4_ in series to form a Si/Al_2_O_3_/ITO/BiVO_4_ photoanode, which will generate sufficient driving force for unbiased water splitting. When coupled to a Pt foil cathode, spontaneous water splitting was realized with an average STH efficiency of 0.62% over a long-term stability test up to 1045 h. This tandem cell is predicted to produce more than 200 mL cm^−2^ of hydrogen, standing out among representative dual-absorber PEC tandem cells for unbiased water splitting.

## Results and discussion

### Construction of an integrated n-Si/BiVO_4_ photoanode with an Al_2_O_3_/ITO interfacial bi-layer

**Fig. 1:**
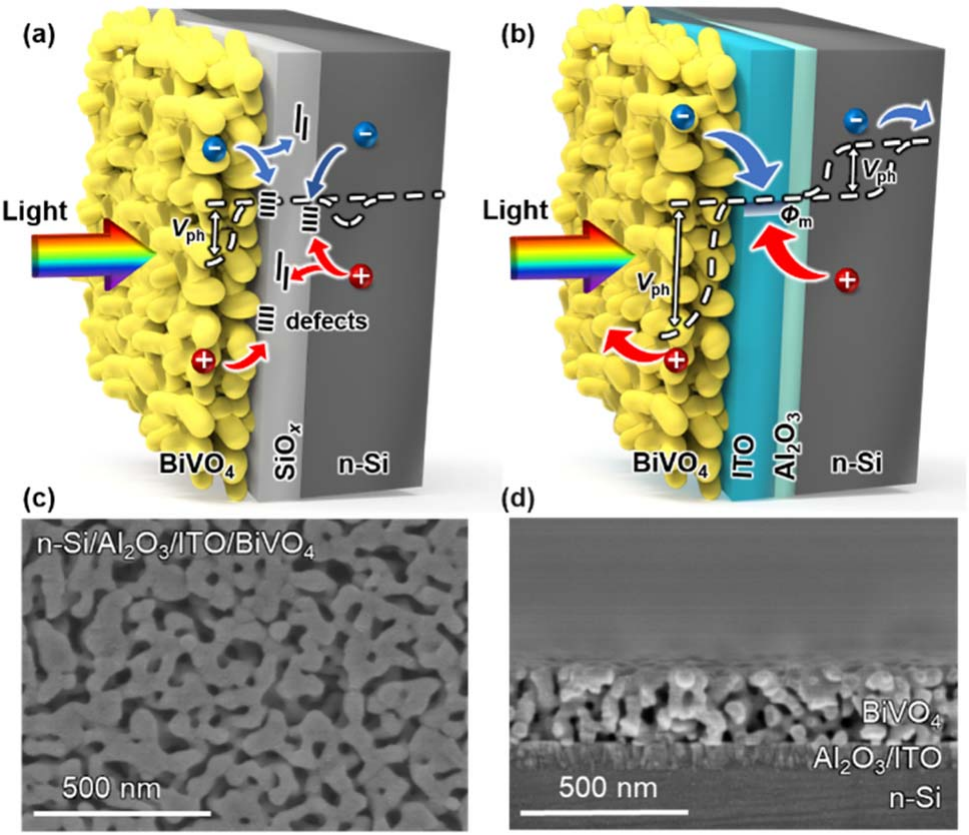
.The schematics of (a) n-Si/SiO_*x*_/BiVO_4_ and (b) n-Si/Al_2_O_3_/ITO/BiVO_4_ integrated tandem photoanodes. The scanning electron microscopy images of (c) the surface morphology and (d) cross sections of the n-Si/Al_2_O_3_/ITO/BiVO_4_ integrated tandem photoanode.

To achieve overall water splitting, several factors need to be considered for the n-Si/BiVO_4_ photoanode: (1) sufficient photovoltage for carrier separation, (2) low-defect interfaces for carrier transfer, and (3) an effective co-catalyst for charge injection. Thus, in order to alleviate the carrier transport problem at a traditional n-Si/BiVO_4_ interface (n-Si/SiO_*x*_/BiVO_4_, Fig. 1a), an Al_2_O_3_/ITO interfacial bi-layer was inserted to form an integrated tandem photoanode (n-Si/Al_2_O_3_/ITO/BiVO_4_, Fig. 1b). The Al_2_O_3_ interfacial layer with an optimized thickness of 2.5 nm was deposited on an n-Si substrate by atomic layer deposition (ALD). The ALD route allows for a conformal coating of Al_2_O_3_ with sub-nanometer precision,^42,43^ which allows for uniform protection of the Si surface during the subsequent annealing process in air avoiding further formation of SiO_*x*_ that hinders carrier transfer. The Al_2_O_3_ interfacial layer also plays a key role in passivating the interfacial defects that prevents carrier recombination, while ensuring the effective tunnelling of carriers at the n-Si/ITO interface.^44^ The ITO interfacial layer with a smooth surface (Fig. S1a†) and an optimized thickness of 80 nm (Fig. S1b†) was deposited on Al_2_O_3_ by radio frequency (RF) sputtering, which serves as a high work function metallic layer to establish a Schottky barrier with n-Si to form an MIS junction that enhances carrier separation,^45,46^ as well as forming an ohmic contact with BiVO_4_ to promote electron transport.^47^

The BiVO_4_ film is deposited on the ITO surface *via* metal–organic decomposition.^48^ Thus, n-Si and BiVO_4_ could be tandemly integrated into an n-Si/Al_2_O_3_/ITO/BiVO_4_ photoanode with the Al_2_O_3_/ITO interfacial bi-layer, where light should first pass through the BiVO_4_ (front illumination) due to the wider band gap of BiVO_4_ than that of n-Si. The n-Si/Al_2_O_3_/ITO MIS junction shows high temperature tolerance at the established annealing temperature for BiVO_4_ (>400 °C), while other buried solid-state junctions developed for solar cells (such as thin film amorphous silicon (a-Si) and heterojunctions with intrinsic thin layer (HIT) Si solar cells) would be damaged at temperatures above 300 °C.^49^ Moreover, the direct integration of a BiVO_4_ absorber with other widely investigated junction structures for solar cells, such as passivated emitter and rear cell (PERC), passivated emitter and rear totally diffused cell (PERT), and interdigitated back contact (IBC), may face additional challenges due to structural incompatibility^50^ (Fig. S2†). In order to obtain the maximum photocurrent under front illumination, the thickness and grain size of the BiVO_4_ film were optimized by adjusting the spin coating speed (Fig. S3†) and annealing conditions (Fig. S4†).^51^ As a result, the n-Si/Al_2_O_3_/ITO/BiVO_4_ tandem photoanode reaches a high PEC performance, where the thickness of the BiVO_4_ film is approximately 250 nm (Fig. 1d) with an average grain diameter of 50 nm (Fig. 1c). For comparison, a BiVO_4_ film was fabricated on a FTO glass substrate (FTO layer deposited on a 2.2 mm glass substrate) to form a FTO/BiVO_4_ photoanode using the same method. The BiVO_4_ exhibits a considerable light transmittance (∼60%) on the FTO glass substrate in the 500–800 nm region (Fig. S5a†), making it suitable as the top absorber to integrate with an n-Si bottom absorber. According to the light transmittance and reflectance (Fig. S5a and b†), the light absorbance of BiVO_4_ can be calculated (Fig. S5c†). Assuming that the absorbed photon-to-current conversion efficiency (APCE) is 100%, the theoretical maximum photocurrent density^48^ (*J*_abs_) for the BiVO_4_ photoanode is 3.2 mA cm^−2^ (Fig. S5d†), which limits the maximum photocurrent density of the n-Si/Al_2_O_3_/ITO/BiVO_4_ tandem photoanode. To further improve the overall PEC performance, strategies such as doping and heterojunction formation can be adopted for BiVO_4_.^21^

### Al_2_O_3_/ITO interfacial bi-layer for improved PEC performance

**Fig. 2:**
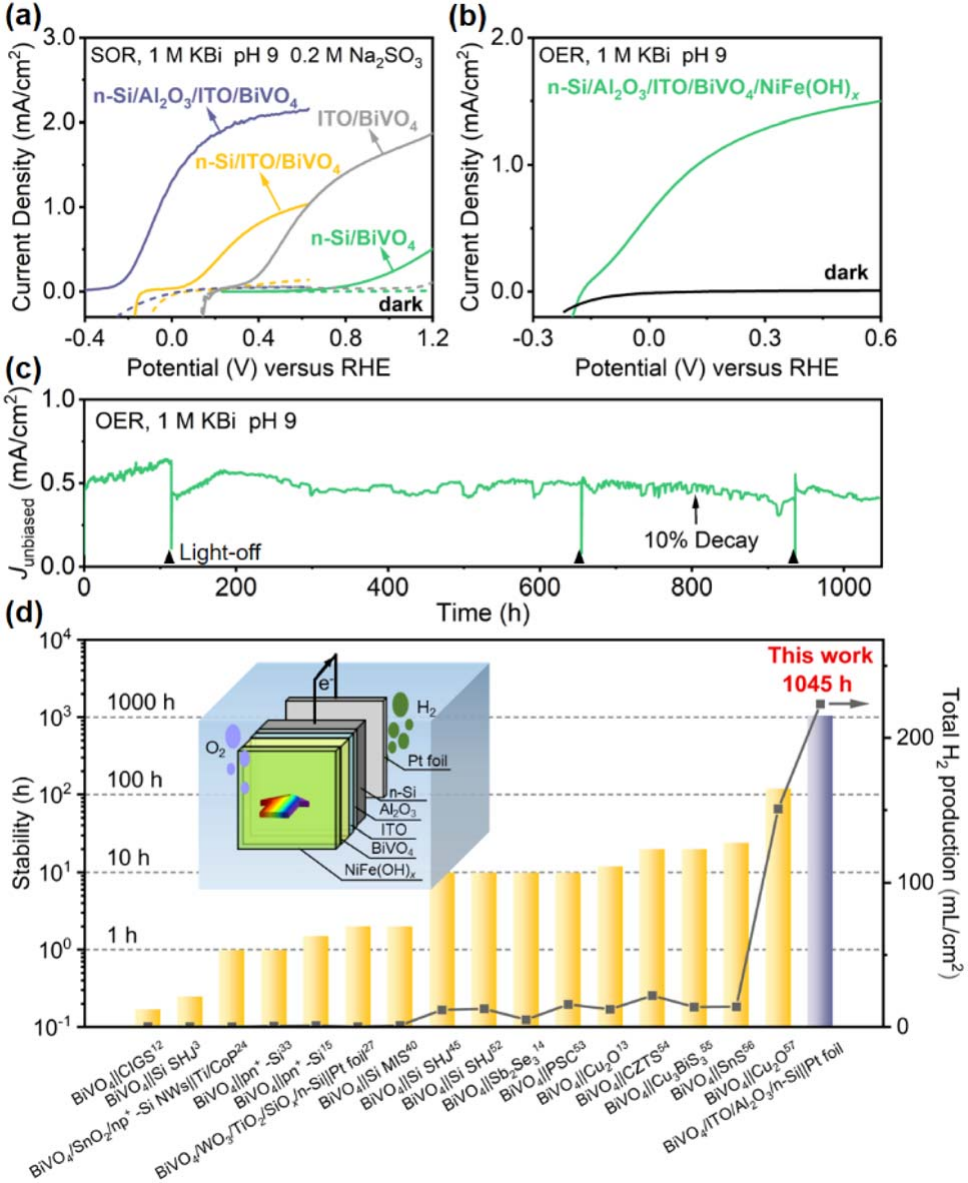
.(a) PEC SOR *J*–*V* curves of n-Si/SiO_*x*_/BiVO_4_, n-Si/ITO/BiVO_4_, ITO/BiVO_4_, and n-Si/Al_2_O_3_/ITO/BiVO_4_ photoanodes. (b) PEC OER *J*–*V* curves and (c) stability test without external bias of an n-Si/Al_2_O_3_/ITO/BiVO_4_/NiFe(OH)_*x*_ integrated tandem photoanode under simulated AM 1.5 G illumination. Light-off was due to lab facility maintenance. (d) Summary of the recently reported stability and the predicted total H_2_ production (over the stable duration) of various representative PEC tandem cells for unbiased water splitting.^3,12–15,24,27,33,40,48,52–57^ Inset: schematic of the tandem water splitting cell consisting of the integrated photoanode and a dark Pt cathode.

To evaluate the effect of this Al_2_O_3_/ITO interfacial bi-layer on PEC performance, the current density–potential (*J*–*V*) curves of the photoanode with or without the Al_2_O_3_/ITO interfacial bi-layer inserted between n-Si and BiVO_4_ were recorded in 1.0 M potassium borate buffer solution (KBi, pH 9.0) containing 0.2 M Na_2_SO_3_ under simulated air mass (AM) 1.5 G sunlight illumination (Fig. 2a). Na_2_SO_3_ is used as a hole scavenger to rule out the effect of sluggish surface reaction kinetics,^48^ and thus the *J*–*V* curve recorded under sulfite oxidation reaction (SOR) conditions is able to estimate the optimal performance of the photoanode using the same material. The n-Si/Al_2_O_3_/ITO/BiVO_4_ photoanode exhibits an early onset potential (defined as the potential required to achieve a photocurrent of 0.1 mA cm^−2^) at −0.25 V *vs.* RHE, with a large photocurrent density (1.3 mA cm^−2^) at 0 V *vs.* RHE as well as a saturation photocurrent density of 2.2 mA cm^−2^, reaching 67% of its theoretical maximum photocurrent density. The n-Si/Al_2_O_3_/ITO electrode shows negligible dark current, indicating no current leakage at the interface between the ITO and the porous BiVO_4_ film (Fig. S6†). The n-Si/Al_2_O_3_/ITO/BiVO_4_ photoanode displays an increase in photovoltage of up to 1000 mV compared to the Si electrode without the Al_2_O_3_/ITO interfacial bi-layer, as evidenced by the difference in onset potential. It is speculated that the Al_2_O_3_/ITO interfacial bi-layer inserted between n-Si and BiVO_4_ may create a large band offset at the n-Si/BiVO_4_ interface as well as repairing the interfacial defect states, providing the driving force for carrier transport.

To demonstrate the practical application of this integrated n-Si/Al_2_O_3_/ITO/BiVO_4_ photoanode for the PEC oxygen evolution reaction (OER), a NiFe(OH)_*x*_ co-catalyst was deposited on the BiVO_4_ surface using a dip coating method, which provides active sites to efficiently utilize the photogenerated carrier for the OER.^58^ The *J*–*V* curve of the n-Si/Al_2_O_3_/ITO/BiVO_4_/NiFe(OH)_*x*_ photoanode in 1.0 M KBi (pH 9.0) demonstrates an onset potential at −0.15 V *vs.* RHE with a photocurrent density of ≈0.6 mA cm^−2^ at 0 V *vs.* RHE and a saturation photocurrent density of 1.5 mA cm^−2^, 48% of its theoretical maximum photocurrent density (Fig. 2b). The charge injection efficiency (*Φ*_ox_) of the NiFe(OH)_*x*_-modified photoanode reaches 70% at 0.6 V *vs.* RHE (Fig. S7a†), which means that the photocurrent under OER conditions reaches 70% of the photocurrent under SOR conditions without the catalyst, indicating the effectiveness of NiFe(OH)_*x*_ in enhancing charge transfer. However, some of the surface-reaching holes were lost due to surface recombination, which can be suppressed by passivation. Upon illumination, spontaneous water splitting was observed when the photoanode was coupled to a Pt foil cathode (inset of Fig. 2d) with notable oxygen bubble generation (ESI Movie†). The accumulation and detachment of bubbles on the electrode surface, the oxidation of Ni and Fe species to the more transparent NiFe(OH)_*x*_ with higher catalytic activity,^59^ and the enriched oxygen vacancies that enhance the charge separation of BiVO_4_ during PEC water oxidation^48^ may be responsible for the fluctuation of the photocurrent (Fig. 2c) (details in the ESI†).

The stability is a major concern for practical solar water splitting systems. Although hundreds of hours of stability have been reported for single photoelectrodes based on BiVO_4_ (ref. 60) or Si,^40^ the stability of Si/BiVO_4_ tandem cells for previous unbiased water splitting is only a few tens of hours (Fig. 2d and Table S1†). The tandem photoanode exhibits a robust photocurrent density with only a 10% drop after 800 h operation. An average photocurrent density of 0.51 mA cm^−2^, equivalent to an STH of 0.62%, is obtained in the 1045 h stability test (Fig. 2c). The weak dark current could be attributed to the rapid switching of light between off and on so that charge stored in BiVO_4_ cannot transfer immediately, which will decrease with prolonged time (Fig. S8†).^61^ The stable operation of the tandem photoanode indicates that the side reactions are well suppressed. Thus, it can be assumed that almost all the photo-generated electrons are consumed for H_2_ production.^40^ To further illustrate the practical competitiveness of the system, the H_2_ production is predicted, which is determined by the efficiency, stability and area of the photoelectrodes.^7,62^ Over 200 mL H_2_ per cm^2^ is expected to be produced during this long-term duration, much more than other representative PEC tandem cells produce in unbiased water splitting (Fig. 2d and Table S1†). The surface morphology is nearly unchanged after the stability test (Fig. S9†). According to the *J*–*V* curves before and after the stability test (Fig. S10†), the photocurrent density at 0 V *vs.* RHE only shows a decay of 19%. The deactivation is likely caused by the inherent instability of BiVO_4_ due to V^5+^ dissolution, which can be prevented by dissolving vanadium cations in borate buffer.^60^ The onset potential, photocurrent density at 0 V *vs.* RHE, and stability are superior to those obtained from all previously reported Si/metal oxide integrated tandem photoanodes (Table S2†).

### Enhanced driving force for carrier transport from the Al_2_O_3_/ITO bi-layer

In order to achieve spontaneous water splitting, the n-Si/BiVO_4_ photoelectrode must provide sufficient driving force to satisfy the thermodynamic potential and kinetic overpotential required to split water. The overall driving force (*i.e.*, photovoltage) for PEC water splitting from the n-Si/BiVO_4_ tandem photoanode is determined by the band offsets at two interfaces: (1) n-Si/interfacial layer and (2) interfacial layer/BiVO_4_. It is crucial to construct an interfacial layer that maximizes the band offsets of the two interfaces, while ensuring low-resistance transport of carriers. In order to clearly demonstrate the contribution of the Al_2_O_3_/ITO interfacial bi-layer for photovoltage generation, PEC SOR analysis of the ITO/BiVO_4_ photoanode and n-Si/Al_2_O_3_/ITO/BiVO_4_ photoanode was performed (Fig. 2a). The ITO/BiVO_4_ photoanode exhibits an onset potential of 0.28 V *vs.* RHE, which agrees well with the adoption of ITO as the ohmic contact for BiVO_4_,^63,64^ suggesting an efficient downward band bending of BiVO_4_ upon the insertion of the ITO interfacial layer. The cathodic shift in the onset potential of the n-Si/Al_2_O_3_/ITO/BiVO_4_ photoanode compared to the ITO/BiVO_4_ photoanode signifies an additional 530 mV photovoltage provided by the n-Si/Al_2_O_3_/ITO MIS junction. The large photovoltage generated by the original MIS junction is attributed to the large barrier height (*Φ*_m_ − *χ*_s_, 1.0 eV) between n-Si (*χ*_s_ ∼ 4.05 eV) and ITO (*Φ*_m_ ∼ 5.05 eV^45,46^). Moreover, the high light transmittance of the ITO layer (Fig. S11†) allows the adoption of a thick layer (80 nm) to establish a fully developed Schottky barrier, which leads to a more profound band bending.

**Fig. 3:**
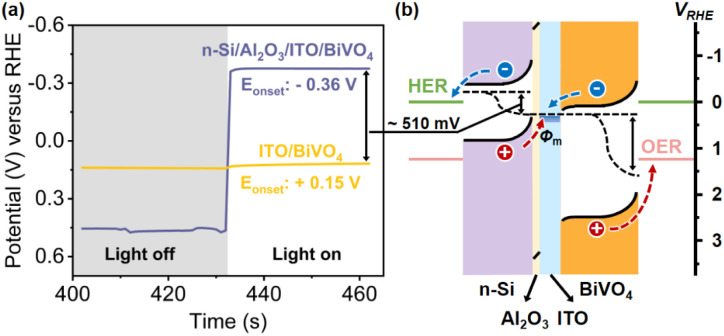
.(a) Dark and AM 1.5 G illuminated time-dependent OCP measurements of the ITO/BiVO_4_ and n-Si/Al_2_O_3_/ITO/BiVO_4_ photoanodes, indicating that the n-Si/Al_2_O_3_/ITO provides an additional 510 mV of photovoltage and the SOR photocurrent onset for the ITO/BiVO_4_ and n-Si/Al_2_O_3_/ITO/BiVO_4_ photoanodes was at 0.15 and −0.36 V *vs.* RHE, respectively. (b) The illustration of the band diagram of the n-Si/Al_2_O_3_/ITO/BiVO_4_ photoanode under illumination.

The difference between the illuminated open circuit potentials (OCPs) of ITO/BiVO_4_ and n-Si/Al_2_O_3_/ITO/BiVO_4_ photoanodes further demonstrates the additional photovoltage provided by the n-Si/Al_2_O_3_/ITO MIS junction, which is measured to be 510 mV (Fig. 3a), consistent with the cathodic shift of the onset potential observed in the *J*–*V* curves (Fig. 2a). Moreover, the OCP (−0.36 V *vs.* RHE) of the n-Si/Al_2_O_3_/ITO/BiVO_4_ photoanode under illumination (the same as the onset potential in Fig. 2a) is more negative than the potential of the HER, indicating that electrons can be extracted to drive the proton reduction reaction. Thus, the integrated photoanode could generate sufficient photovoltage to achieve water splitting without external bias.

The improvement of the photovoltage after the adoption of the Al_2_O_3_/ITO interfacial bi-layer is illustrated in the energy band diagrams of the n-Si/Al_2_O_3_ (ref. 63 and 64)/ITO/BiVO_4_ photoanode (Fig. 3b). With the Al_2_O_3_/ITO interfacial bi-layer, both the upward band bending of n-Si and downward band bending of BiVO_4_ are satisfied at the interface, which maximizes the interfacial band offset between n-Si and BiVO_4_, producing sufficient photovoltage for unassisted water splitting. Under these conditions, carrier transport at the interface (electrons from BiVO_4_ recombining with the holes from n-Si) as well as carrier separation within the double absorbers are promoted.

### Suppressed interfacial carrier recombination from the Al_2_O_3_/ITO bi-layer

Another important factor that determines the overall performance of a photoelectrode is the interfacial carrier recombination, which could become increasingly severe when introducing additional interfaces. Compared with the relatively simple ITO/BiVO_4_ photoanode, the n-Si/ITO/BiVO_4_ photoanode presents a lower saturation photocurrent density and a gentler slope of the *J*–*V* curve (Fig. 2a) upon the introduction of the additional Si/ITO interface, despite the large interfacial band offset provided by n-Si/BiVO_4_. This unexpected poor performance of the n-Si/ITO/BiVO_4_ photoanode is mainly attributable to the undesired defect states at the n-Si/ITO interface that hinders carrier transport.

To fully utilized the band offset between n-Si/ITO and ITO/BiVO_4_ interfaces, while preventing the negative effect of interfacial defects, 2.5 nm Al_2_O_3_ was inserted at the n-Si/ITO interface by ALD, which leads to a significant increase in the saturation photocurrent density and slope for the *J*–*V* characteristics of n-Si/ITO/BiVO_4_, comparable to that of the ITO/BiVO_4_ photoanode (Fig. 2a). This enhancement can be attributed to the surface passivation of the Al_2_O_3_ interfacial layer that prevents carrier recombination^65^ while ensuring the tunnelling of carriers.^66^ The Al_2_O_3_ interfacial layer shows high light transmittance (Fig. S11†) due to its large band gap (6.7 eV)^67^ and thin thickness (2.5 nm), while other layers such as HfO_2_ (5.8 eV),^68^ ZrO_2_ (4.9 eV)^69^ and TiO_2_ (3.3 eV)^70^ may also be applicable if they could be deposited with a suitable structure for defect passivation within a thickness thin enough. Moreover, the PEC performance of the n-Si/Al_2_O_3_/ITO/BiVO_4_ tandem photoanode can be adjusted by controlling the Al_2_O_3_ thickness (Fig. S12†). If the thickness is too thin (<1.5 nm), Al_2_O_3_ will not provide sufficient surface passivation, while the carrier transport resistance will increase significantly if the Al_2_O_3_ is too thick (>3.5 nm).^71,72^

**Fig. 4:**
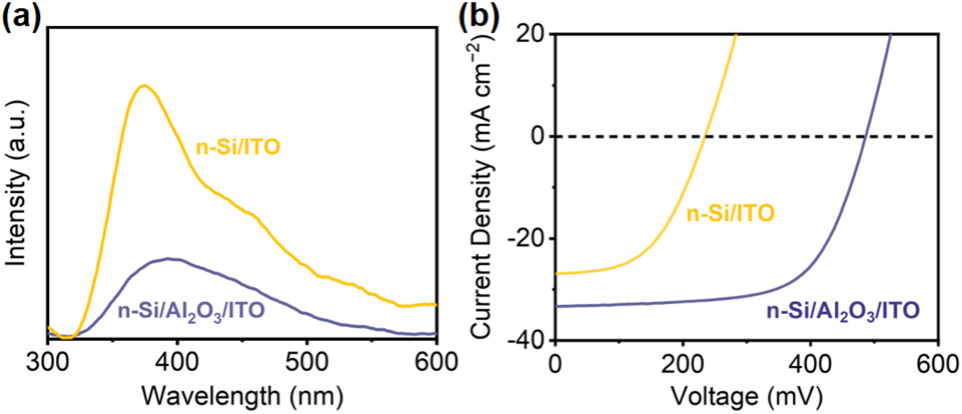
.(a) PL spectra and (b) solid-state *J*–*V* characteristics of n-Si/ITO and n-Si/Al_2_O_3_/ITO.

To evaluate the passivation of Al_2_O_3_, steady-state photoluminescence spectroscopy (PL) was conducted to examine charge recombination^73,74^ (Fig. 4a). n-Si/ITO exhibits strong fluorescence intensity, which implies a severe charge recombination. Upon Al_2_O_3_ passivation, the intensity weakened, indicating that the defects at the n-Si/ITO interface are effectively eliminated. The passivating effect of Al_2_O_3_ can be further demonstrated by the minority carrier lifetime, which is improved from 5.79 μs for n-Si to 8.31 μs for n-Si/Al_2_O_3_. The corresponding surface recombination velocity of the Si substrate (1295 cm s^−1^) is reduced to 902 cm s^−1^ with passivation.^3^ Moreover, the influence of the Al_2_O_3_ interfacial layer on the MIS junction was analyzed by comparing the *J*–*V* curves of the n-Si/ITO and n-Si/Al_2_O_3_/ITO solid-state devices. The solid-state n-Si/Al_2_O_3_/ITO device shows a higher PEC performance than that obtained from the n-Si/ITO device (Fig. 4b), indicating that the Al_2_O_3_ interfacial passivation layer could effectively improve the carrier transport of the Si MIS junction.

Therefore, the enhanced PEC performance of the n-Si/Al_2_O_3_/ITO/BiVO_4_ tandem photoanode was found to be partly due to the high-quality passivation of the Al_2_O_3_ interlayer achieving sufficient interfacial carrier transport. As a result, upon the introduction of the ITO interfacial layer, the overall charge separation efficiency (*Φ*_sep_) of the n-Si/BiVO_4_ photoanode increases from 0.4% to 31.8% at 0.6 V *vs.* RHE (Fig. S7b†), which can be attributed to the favorable energy band position of ITO that allows the creation of a Schottky junction and ohmic contact to largely enhance interfacial charge separation. After the employment of the Al_2_O_3_ interfacial layer, the *Φ*_sep_ further increases to 66.7% due to its outstanding passivation effect in the newly developed n-Si MIS junction. In this way, the Al_2_O_3_/ITO interfacial bi-layer between n-Si and BiVO_4_ enhances the driving force for carrier transport while suppressing the interfacial carrier recombination, breaking through the long-standing trade-off between photovoltage generation and interfacial defect passivation of conventional n-Si/BiVO_4_ integrated photoelectrodes.

**Fig. 5:**
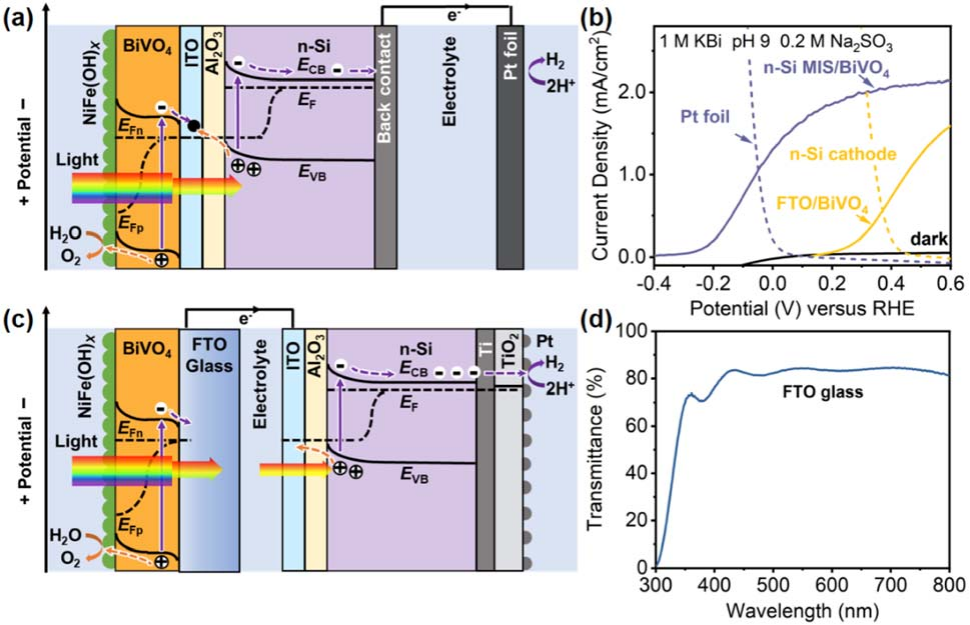
.Schematic energy band diagrams of (a) the n-Si/Al_2_O_3_/ITO/BiVO_4_ integrated tandem photoanode and dark Pt cathode and of (c) the BiVO_4_ photoanode and n-Si photocathode tandem water splitting cell. *E*_CB_ is the conduction band edge, *E*_VB_ is the valence band edge and *E*_F_ is the Fermi level. Layer thickness is not to scale, for clarity purposes. (b) PEC SOR *J*–*V* curves of different tandem cells under simulated AM 1.5 G illumination. (d) UV-vis transmission spectra of the FTO glass substrate.

To illustrate the advantage of this integrated Si/BiVO_4_ photoelectrode (Integ. PEC) over a separated tandem system composed of a Si-based photocathode (or solar cell) and BiVO_4_-based photoanode (PEC/PEC or PV/PEC systems), both integrated and separated tandem cells were fabricated to compared their PEC performances. In the integrated case, an n-Si/Al_2_O_3_/ITO/BiVO_4_ photoanode (integrated photoelectrode) is coupled to a dark Pt foil cathode (Fig. 5a). In the separated case, a BiVO_4_ photoanode and n-Si MIS photocathode (or a solar cell) are fabricated independently and connected *via* wires (Fig. 5c and S13a†). The Integ. PEC system obtains a higher unbiased current density of 1.05 mA cm^−2^ compared to the separated photoelectrodes (0.56 and 0.98 mA cm^−2^ for the PEC/PEC and PV/PEC systems, respectively) (Fig. 5b and S13b†). The corresponding Tafel slope of the Pt foil and sputtered Pt layer on the n-Si photocathode is 137 and 131 mV dec^−1^ (Fig. S14†), implying the same HER kinetics. Thus, the higher PEC performance may be due to the mitigation of optical scattering losses (>20%) caused by the FTO glass (Fig. 5d), which allows Si to absorb more light to increase the driving force for water splitting, *i.e.* 0.53 V generated by the n-Si/Al_2_O_3_/ITO junction (Fig. 2a), which is higher than that of the n-Si photocathode (0.45 V, Fig. 5b). Moreover, the elimination of ohmic losses caused by the FTO/wire/ITO interfaces will further promote the charge transfer.^75^ The effectiveness after the integration of n-Si and BiVO_4_ is further illustrated in the energy band diagrams of the integrated (Fig. 5a) and separated (Fig. 5c and S13b†) photoelectrodes. For the integrated photoelectrodes (Fig. 5a), n-Si could adsorb a significant portion of incident light for carrier generation, which is attributed to the elimination of optical losses caused by FTO glass as well as the electrolyte. The Al_2_O_3_/ITO interfacial bi-layer integrates n-Si and BiVO_4_ into a single photoelectrode, where ITO forms a Schottky junction and an ohmic contact with n-Si and BiVO_4_ simultaneously, while Al_2_O_3_ suppresses the carrier recombination, thus maximizing the interfacial band offset to generate photovoltage for enhanced carrier separation. Hence, more photogenerated carriers can be injected into the water redox reaction. Although the separated case is prototyped by mechanically combining the two photoelectrodes (Fig. 5c and S13b†), the parasitic optical loss caused by the FTO glass is unavoidable, resulting in less light absorption by n-Si for carrier generation. In terms of cost, the integrated photoelectrode combined BiVO_4_ and n-Si into a single monolithic electrode without the use of FTO glass, providing sufficient flexibility at a lower fabrication complexity and cost.

## Conclusions

This work designs and fabricates an integrated n-Si/BiVO_4_ photoanode using an Al_2_O_3_/ITO interfacial bi-layer to facilitate carrier transport for unbiased solar water splitting. Specifically, the ITO layer is introduced to realize the simultaneous formation of a Schottky junction and an ohmic contact at the n-Si/BiVO_4_ interface to create additional band offsets for n-Si and BiVO_4_, which could provide sufficient driving force for carrier transport. Furthermore, an Al_2_O_3_ interfacial passivation layer is inserted to heal the defects at the newly formed n-Si/ITO interface, through which the carrier recombination can be suppressed to allow unimpeded carrier transport. The adoption of this Al_2_O_3_/ITO interfacial bi-layer enlarges the photovoltage for more than 1000 mV compared to the n-Si/BiVO_4_ photoanode. Upon the loading of the surface co-catalyst, this integrated n-Si/Al_2_O_3_/ITO/BiVO_4_/NiFe(OH)_*x*_ photoanode could achieve spontaneous solar water splitting when coupled to a Pt foil cathode. An impressive 1045 h of stability was obtained with an average STH of 0.62% and the total hydrogen production is predicted to exceed 200 mL cm^−2^. This work provides essential insights for the improvement of interfacial carrier transport in the integration of photoelectrodes, which eliminates the contradiction between interfacial band alignment and defect passivation, advancing the frontier technology of solar water splitting. To make the PEC system competitive, further efficiency and stability improvements are necessary, which requires technological advances to meet material performance targets along with suitable application scenario exploration based on the specific deployment to achieve appropriate plant-scale engineering.

## Data availability

The data that supports the findings of this study is available from the corresponding author upon request.

## Author contributions

S. W. and J. G. supervised the research. S. W., T. W. and J. G. conceived the ideas and designed the experiments. S. W., B. L. and Z. G. conducted the experiments of material synthesis, device fabrication, electrochemical measurements, materials characterization and data analysis. All authors discussed the results and participate in writing the manuscript.

## Conflicts of interest

There are no conflicts to declare.

## Supplementary Material

SC-014-D2SC06651C-s001

SC-014-D2SC06651C-s002
