# Supplementary material for: An integrated n-Si/BiVO4 photoelectrode with an interfacial bi-layer for unassisted solar water splitting
Source: Chem Sci. 2023 Jan 30;14(8):2192–9. doi: 10.1039/d2sc06651c (PMC9945263; doi:10.1039/d2sc06651c)
Supplement: SC-014-D2SC06651C-s001 [file SC-014-D2SC06651C-s001.pdf]

Electronic Supplementary Material (ESI) for Chemical Science.
This journal is © The Royal Society of Chemistry 2023

Supplementary Information

Integrated n-Si/$BiVO_4$ Photoelectrode with an Interfacial Bi-layer for Unassisted Solar Water Splitting

Shujie Wang [a,b], Shijia Feng [a,b], Bin Liu [a,b], Zichen Gong [a,b], Tuo Wang [a,b,c,d,e*] and Jinlong Gong [a,b,c,e*]

[a] School of Chemical Engineering and Technology; Key Laboratory for Green Chemical Technology of Ministry of Education, Tianjin University,Tianjin, 300072, China,

[b] Collaborative Innovation Center of Chemical Science and Engineering (Tianjin), Tianjin 300072, China.

[c] Haihe Laboratory of Sustainable Chemical Transformations, Tianjin 300192, China

[d] Joint School of National University of Singapore and Tianjin University, International Campus of Tianjin University, Binhai New City, Fuzhou 350207, China

[] National Industry-Education Platform of Energy Storage, Tianjin 300350, China

* Corresponding authors: jlgong@tju.edu.cn; wangtuo@tju.edu.cn

Experimental Procedures

Preparation of Si bottom absorber.

Phosphorus-doped (n-type, (100)-oriented, single -side polished, resistivity 0.42–0.86 Ωcm, 150 μm) Si wafers and degenerately boron-doped (p^+-type, (100)-oriented, single-side polished, resistivity 0.001–0.01 Ωcm, 500 μm) Si wafers were used as the bottom absorber. To remove organic contaminants, the Si wafers were first immersed in a piranha solution containing a mixture of sulfuric acid (H_2SO_4, 95.0–98.0%) and hydrogen peroxide (H_2O_2, 30% (w/w) in H_2O, contains stabilizer) with a volume ratio of 3:1. To strip the native oxide SiO_x, the Si wafers were dipped in a 5% hydrofluoric acid (HF, ≥ 40.0%) solution for 1.5 min, followed by rinsing with deionized water and drying with N_2. The SiO_x thickness of the Si wafers was reduced to 0.6–0.8 nm after treatment as measured by spectroscopic ellipsometry (M-2000D, J. A. Woollam).

The deposition of Al_2O_3 interfacial layer.

After cleaning by the piranha solution and HF solution, Al_2O_3 interfacial layers with various thicknesses were deposited on the above Si absorbers at 150 °C in a custom-made atomic layer deposition (ALD) system. Trimethyl aluminum (TMA, 99.999%, Suzhou Fornano Electronics Technology Co., LTD.) and ultrapure H_2O (room temperature) were used as precursors. In a typical process, one ALD cycle consists of TMA dose for 0.04 s, N_2 purge for 5 s, H_2O dose for 0.3 s, and N_2 purge for 5 s. The growth rate of Al_2O_3 was 0.1 nm per cycle.

The deposition of ITO interfacial layer.

An 80 nm thick ITO (In_2O_3: SnO_2 = 90%: 10%, ≥ 99.99%) film was deposited by radio frequency (RF) sputtering. The chamber base pressure is 10^{-5} Pa, during deposition, the Ar flow was introduced into the chamber, with gas flows fixed at 20 sccm, and the working pressure was held at 0.2 Pa. The sputtering power was kept at 40 W and the deposition rate was approximately 3.833 Å s^{-1}. After the deposition, the samples were treated with UV ozone for 15 min at 60 °C, which was found to improve the surface work-function of ITO and enhance the adhesion of the $BiVO_4$ layer[1].

The deposition of $BiVO_4$ top absorber.

The $BiVO_4$ film was deposited on the above Si/Al_2O_3/ITO and FTO (F:SnO_2, 14 Ω per square,) substrates by metalorganic decomposition (MOD) method[2]. Specifically, 0.2425 g bismuth nitrate pentahydrate ($Bi(NO_3)_3 \cdot 5H_2O$, VR) and 0.1325 g vanadyl acetylacetonate ($VO(acac)_2$, 99.0%) were mixed in 500 μL dimethyl sulfoxide (DMSO, AR) and sonicated for 30 min at room

temperature to obtain 1 M precursor solution. Then the precursor solution was spin coated on the Si/Al_2O_3/ITO, ITO and FTO at a substrate temperature of 60 °C. The spin coating speed was optimized to 1000 rpm for 20 s followed by 3000 rpm for 40 s to obtain approximately $BiVO_4$ thickness. In order to obtain compatible annealing conditions for each layer, the annealing condition was optimized to 400 °C for 0.5 h in the air. The obtained electrodes are soaked in 1 M KOH for 10 min to strip the excess VO_x species[3]. A facile photoetching approach was applied to enhance the charge separation by immersing the obtained film in 1 M potassium borate buffer solution (KBi, pH 9.0) containing 0.2 M Na_2SO_3 (98%) under simulated air mass (AM) 1.5 G illumination for 10 min.

The deposition of $NiFe(OH)_x$ catalyst.

For the catalyst, a 20 μL precursor solution containing 30 mM sodium citrate, 5 mM $Fe(NO_3)_3$, and 5 mM $Ni(NO_3)_2$ was dropped cast onto the $BiVO_4$ film and then dried at 60 °C for 1 h. The obtained electrodes are soaked in 1 M KOH for 5 min to covert the complex into hydroxide, followed by rinsing with deionized water and drying with N_2 to remove the excess hydroxide[4].

Photoelectrochemical measurements.

The PEC measurements of the photoanode were performed in a three-electrode configuration using a Hg/HgO reference, and a Pt foil counter electrode. Ga-In alloy (75.5:24.5 wt%, ≥99.99%) was rubbed on the back of Si to form an ohmic contact. The exposed edges were sealed with an epoxy adhesive. Sulfite oxidation reaction (SOR) was performed in 1.0 M potassium borate buffer solution (KBi, pH 9.0) containing 0.2 M Na_2SO_3 under simulated AM 1.5 G sunlight illumination. Oxygen evolution reaction (OER) was performed in 1.0 M KBi (pH 9.0). The measured potentials versus Hg/HgO were converted to the RHE scale, $E_{RHE} = E_{Hg/HgO} + 0.059pH + 0.098$[5].

An electrochemical workstation (CompactStat.e20250, IVIUM) was used to measure the *J-V* curves and chronoamperometry under the irradiation provided by a xenon lamp (PLS-FX300HU, Beijing Perfectlight), equipped with an AM 1.5 G filter. The light intensity was adjusted to 100 mW cm^{-2} against a calibrated Si photodiode (Thorlabs, Inc.). The *J-V* curves were measured at 50 mV s^{-1}. The active areas of the working electrode were determined by the software Image J.

The solar-to-hydrogen (STH) efficiency of the electrodes above was calculated from the *J-V* curves with an assumption of 100% Faradaic efficiency (FE), according to the equation STH = I × V × FE / P [6]. Where I (mA cm^{-2}) is the photocurrent density, V (1.23 V) is the thermodynamic water splitting potential (based on ΔG^0), and P (mW cm^{-2}) is the incident illumination intensity (100 mW cm^{-2} in this work).

The OCP measurement was first performed in the dark. After the potential had stabilized, the light was turned on[7]. The OCP value was the average of more than three different devices.

The sustainable water oxidation performance of the integrated photoanode were conducted without bias. The water oxidation process under illumination tends to oxidize the Ni and Fe species to NiFe(OH)$_x$ with high catalytic activity, as well as enrich the surface oxygen vacancies of $BiVO_4$ that promote carrier separation, which may be responsible for the increase of photocurrent. After first 100 h stability, the test was stopped due to a power outage, the integrated photoanode was left in air without light illumination and bias for 2 days. Part of oxygen vacancies were filled by the oxygen from air during the 2 days, leading to the initial decrease in photocurrent. The second and third light-off duration were short, which shown less effect on the photocurrent.

The H_2 production of the PEC tandem cells in Table S1 were calculated from the stability curves with an assumption of 100% Faradaic efficiency and 1 cm^2 electrode area, according to the ideal gas equation PV=nRT. Where P (Pa) is the pressure of the ideal gas, V (m^3) is the volume of the ideal gas, n (mole) is the amount of ideal gas measured in terms of moles, R is the gas constant (8.314 J K^{-1}mol^{-1}), T (K) is the temperature. FE=αnF/It, where F is Faraday's constant, α is the number of electrons exchanged, I (mA cm^{-2}) is the total current and t (h) the stability.

The J_{abs} (the rate of photon absorption expressed as a current density, which is calculated assuming the absorbed photon-to-current conversion efficiency (APCE) to be 100%) of $BiVO_4$ photoanode was calculated by measuring the light absorbance (A) and integrating it with respect to the AM 1.5G solar spectrum[8], where A= 100%-T-R (T and R is the light transmittance and reflectance investigated by UV-vis spectroscopy, respectively).

The charge separation efficiency (Φ_{sep}) and charge injection efficiency (Φ_{ox}) were calculated from the equations: $J_{PEC} = J_{abs} \times \Phi_{sep} \times \Phi_{ox}$, where J_{PEC} is the measured photocurrent density[8]. For SOR with extremely fast oxidation kinetics, surface recombination is negligible, Φ_{ox} becomes 1, thus $\Phi_{sep}= J_{SOR}/J_{abs}$. Upon the loading of NiFe(OH)$_x$ for OER, the Φ_{ox} can be further calculated.

Characterization.

The top-down and cross-sectional morphologies of the devices were imaged using a field emission scanning electron microscope (FE-SEM, Hitachi S-4800, 5 kV). The thicknesses of the ITO and Al_2O_3 on the polished Si (100) monitor substrate were obtained using a spectroscopic ellipsometer (M-2000 D, J. A. Woollam Co., Inc.) at incident angles of 60° and 70°, by fitting the amplitude ratio (Ψ) and phase shift (Δ) of polarized light with the Cauchy dispersion model for ITO and Al_2O_3. The Si monitor substrate was placed 5 mm next to the Si sample in the same batch. *J-V* curves of the solid-state cells were measured on a source-meter (Model 2450, Keithley

Instruments) in the voltage range from –0.5 to 1.0 V. A Ga-In alloy was rubbed on the Si to make a back contact on the electrodes and Cu wire was connected to the metallic film by silver conductive adhesive. PL spectra were obtained on a Hitachi F-4600 fluorescence spectrophotometer. The transmission spectra were recorded in the range of 300-800 nm at room temperature by a SHIMADZU UV-2550 spectrophotometer. The minority carrier life time (MCLT) was measured with transient photoconductance decay (PCD) using WCT-120 lifetime tester. The surface recombination velocity was calculated by the equation: $1/\tau_{eff}=1/\tau_{bulk} + 2S/W$, where τ_{bulk} is the bulk minority carrier lifetime, W is the thickness of Si, S is the surface recombination velocity[9].

Supplementary Figures

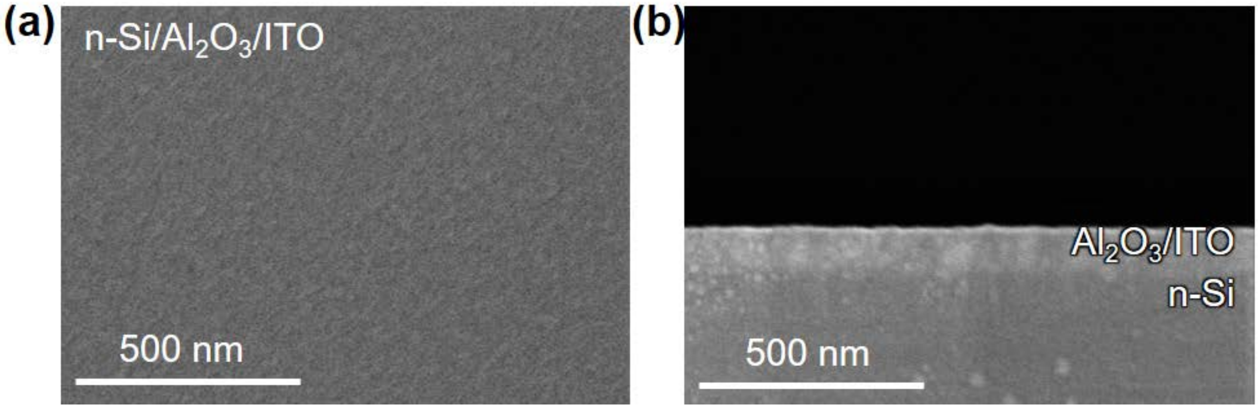

Figure S1. Scanning electron microscopy images of (a) surface morphology, and (b) cross-sections of n-Si/Al_2O_3/ITO.

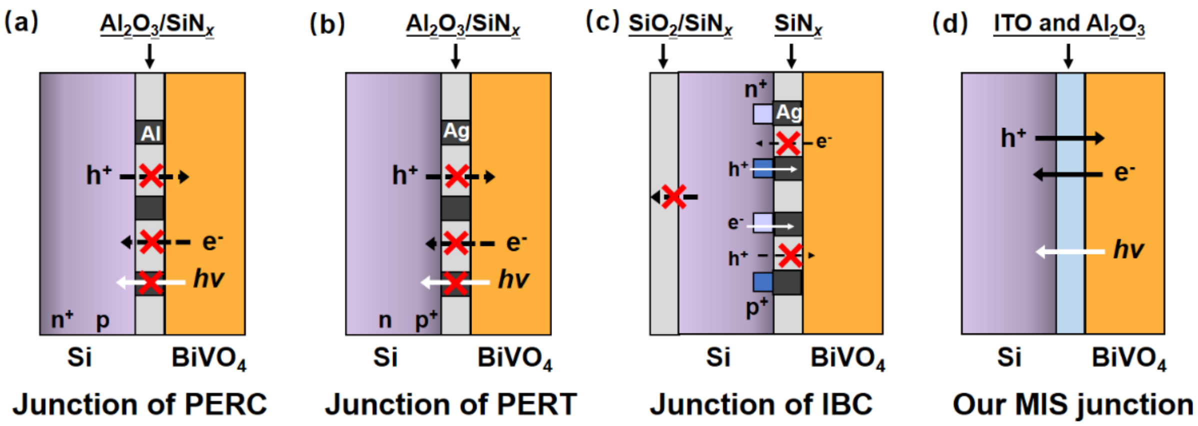

Figure S2. Schematic representation of the integration of typical solid-state junctions with $BiVO_4$. Solid-state junctions of (a) passivated emitter and rear cell (PERC) (b) passivated emitter and rear totally diffused (PERT) cells and (c) interdigitated back contact (IBC) cell, and (d) Si/Al_2O_3/ITO MIS junction for our integrated photoanode. Passivation or anti-reflection layers, such as SiO_2, Al_2O_3 and $SiNi_x$ are insulators through which carriers cannot pass. The metal point contact (Al and Ag) enhances the carrier transport by photolithography, but optical losses, stability issues and additional cost should be considered.

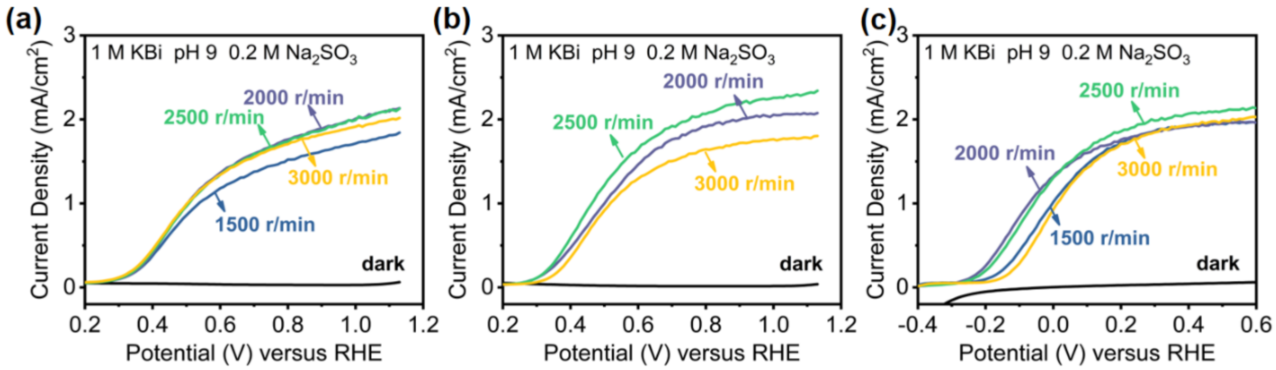

Figure S3. PEC SOR *J*-*V* curves of (a) FTO/$BiVO_4$ photoanodes, (b) p^+-Si/Al_2O_3/ITO/$BiVO_4$ photoanodes, and (c) n-Si/Al_2O_3/ITO/$BiVO_4$ integrated tandem photoanodes with various spin coating speeds of precursor solution.

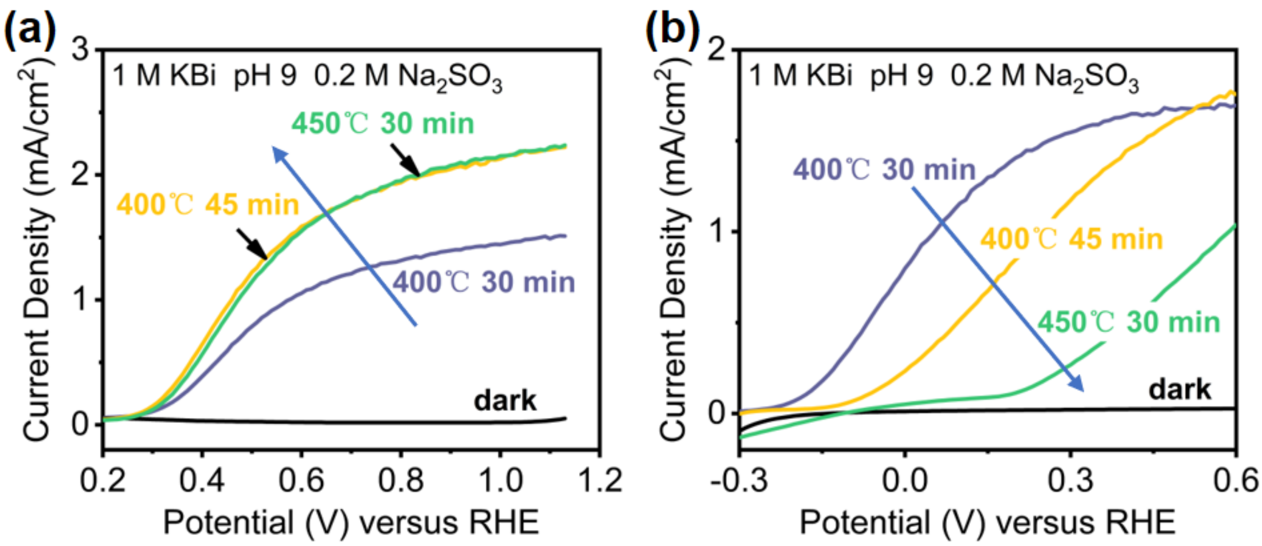

Figure S4. PEC SOR *J-V* curves of (a) FTO/$BiVO_4$ photoanodes, and (b) n-Si/Al_2O_3/ITO/$BiVO_4$ integrated tandem photoanodes with various annealing conditions.

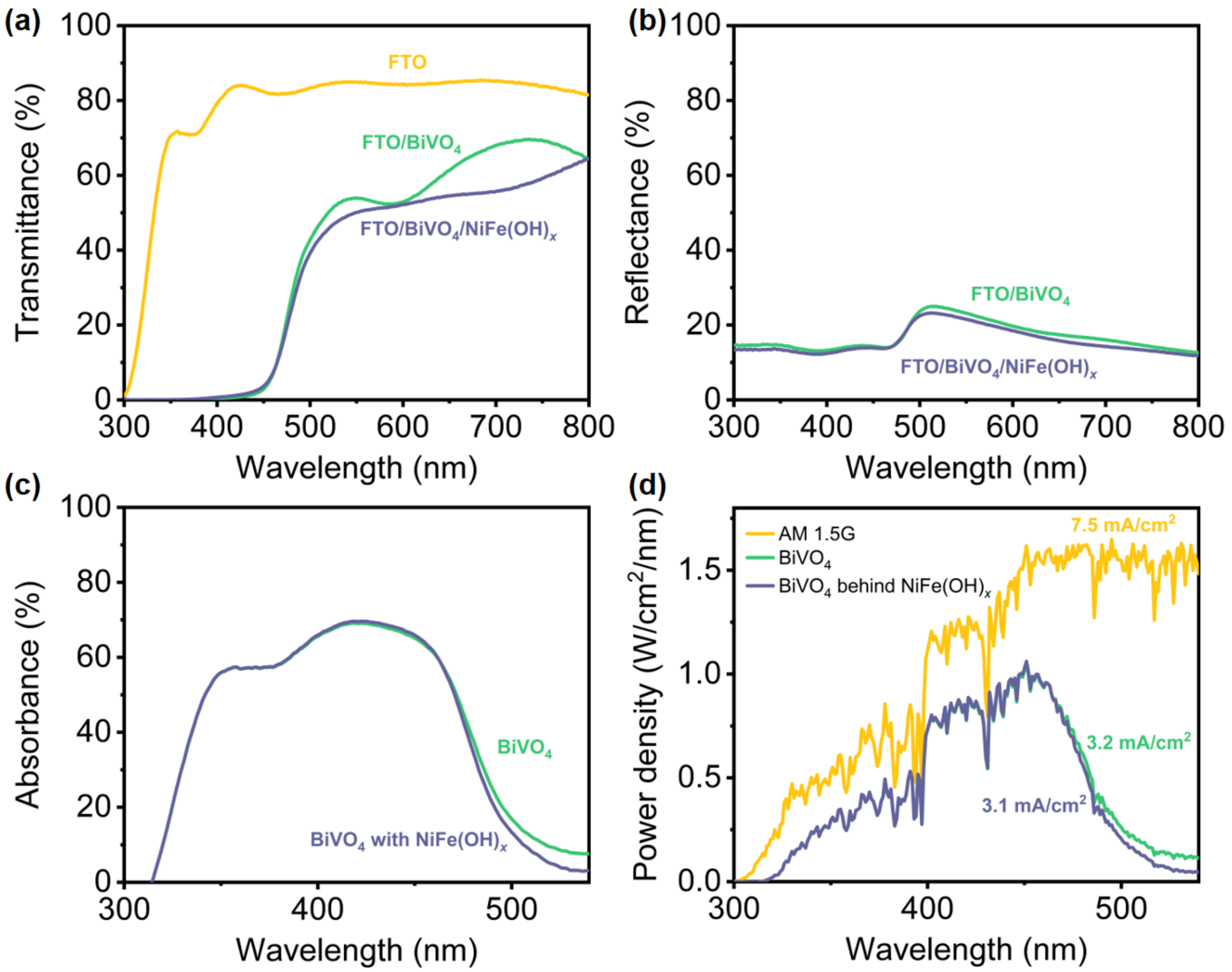

Figure S5. (a) Light transmittance, (b) reflectance, (c) absorbance spectra, and (d) theoretical photocurrent density of $BiVO_4$ and $BiVO_4/NiFe(OH)_x$ photoanodes.

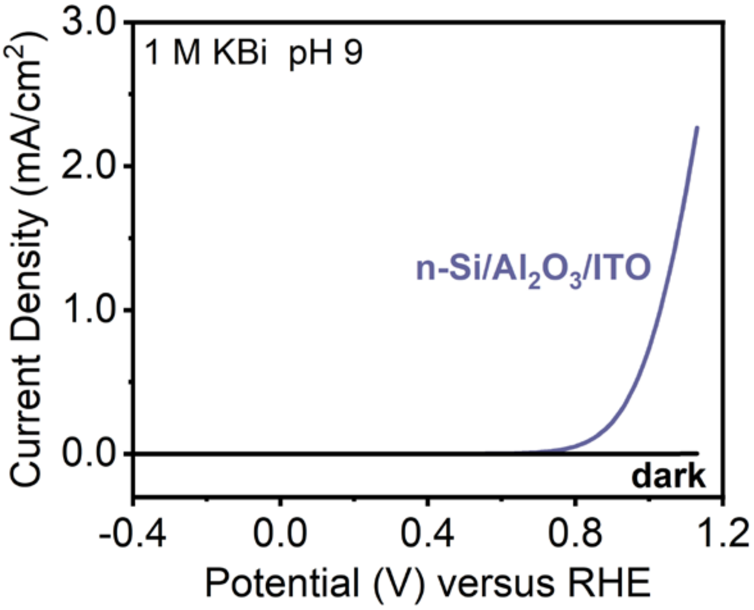

Figure S6. PEC *J*-*V* curves of n-Si/Al_2O_3/ITO photoanode.

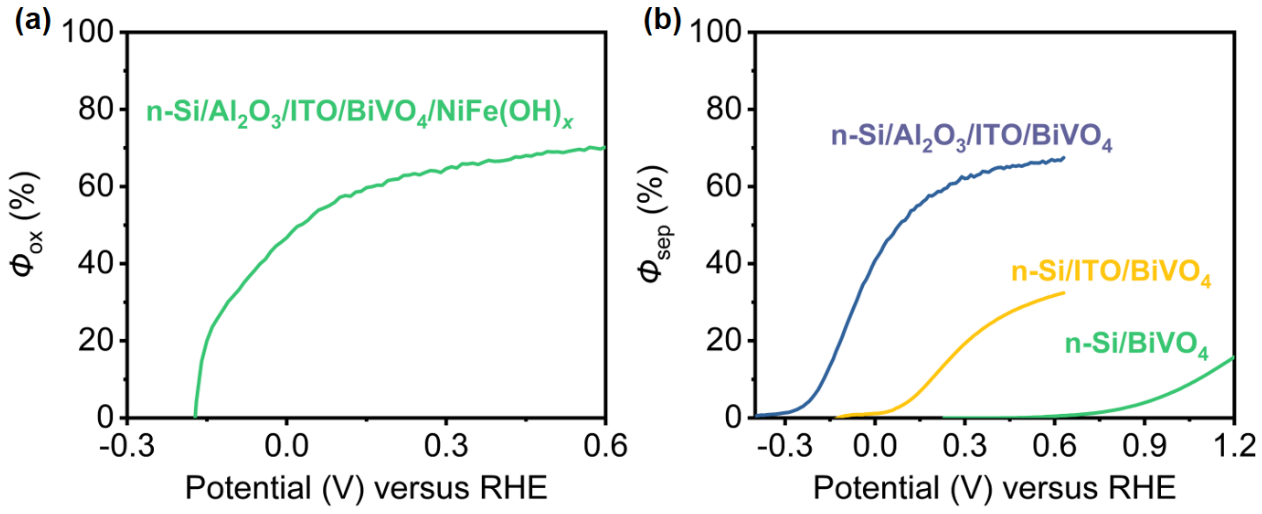

Figure S7. (a) charge injection efficiency of n-Si/Al_2O_3/ITO/$BiVO_4$/NiFe$(OH)_x$ photoanode. (b) charge separation efficiency of n-Si/$BiVO_4$, n-Si/ITO/$BiVO_4$ and n-Si/Al_2O_3/ITO/$BiVO_4$ photoanodes.

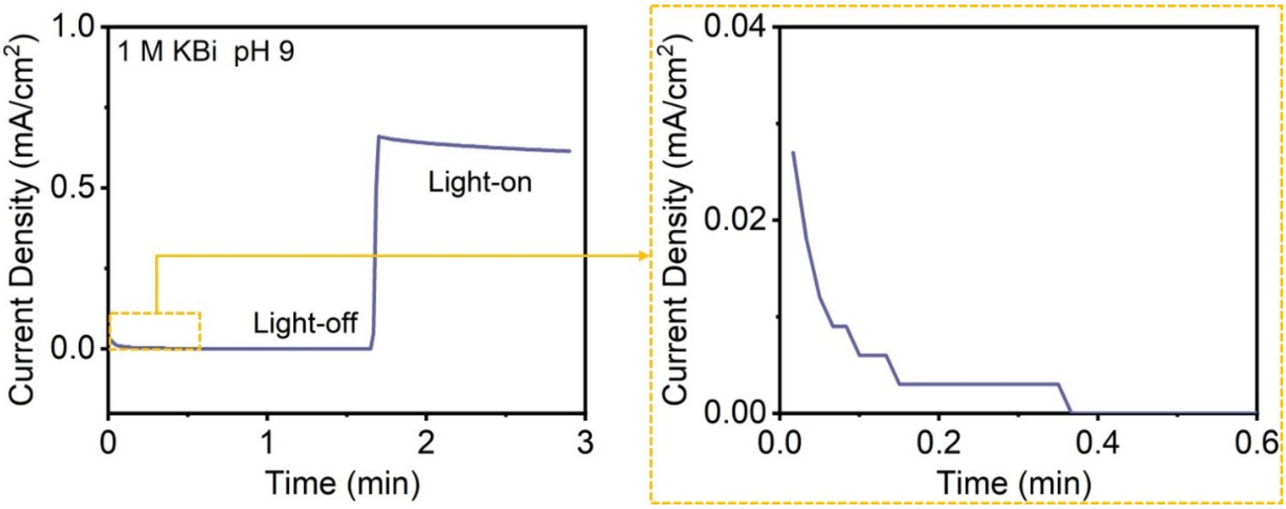

Figure S8. Transient photocurrent measurements of n-Si/Al_2O_3/ITO/$BiVO_4$ photoanode.

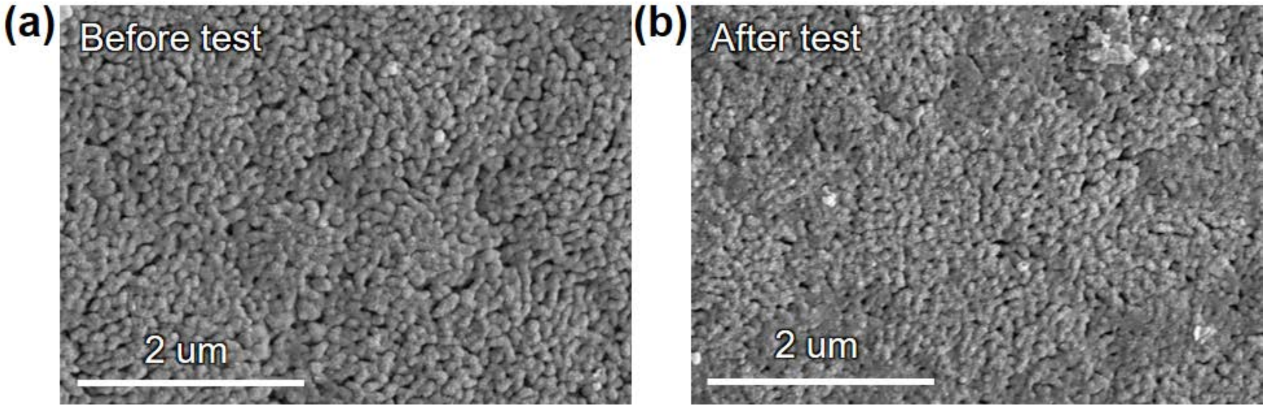

Figure S9. Scanning electron microscopy images of n-Si/Al_2O_3/ITO/$BiVO_4$/NiFe$(OH)_x$ photoanode (a) before and (b) after 1045-h stability test.

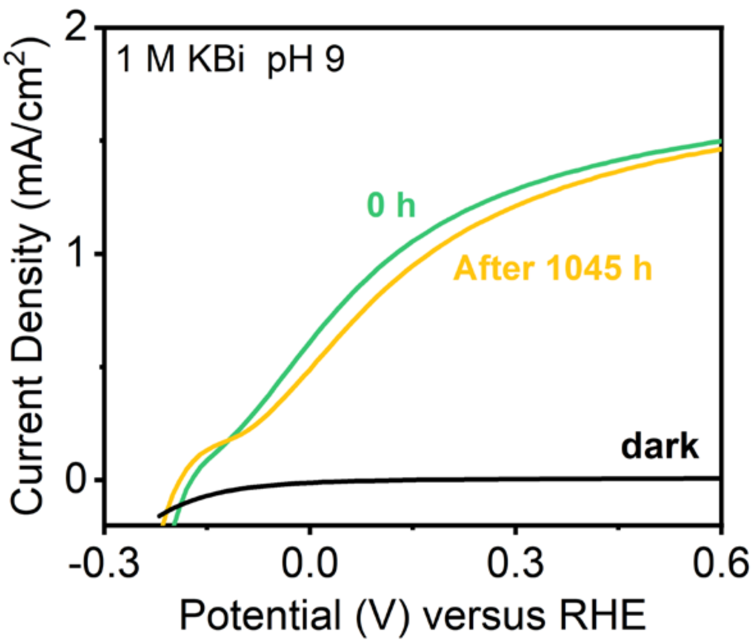

Figure S10. *J*-*V* curves of n-Si/Al_2O_3/ITO/$BiVO_4$/NiFe$(OH)_x$ photoanode before and after 1045-h stability test.

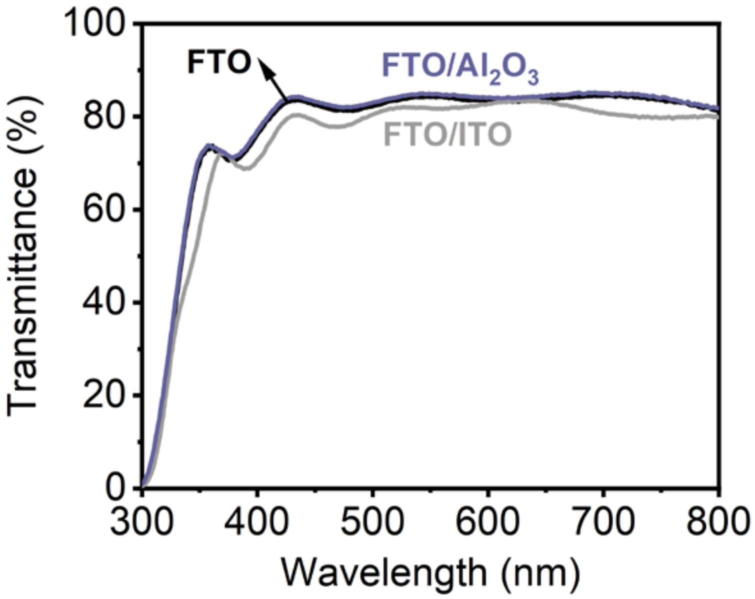

Figure S11. UV-vis transmission spectra of FTO/ITO and FTO/Al_2O_3.

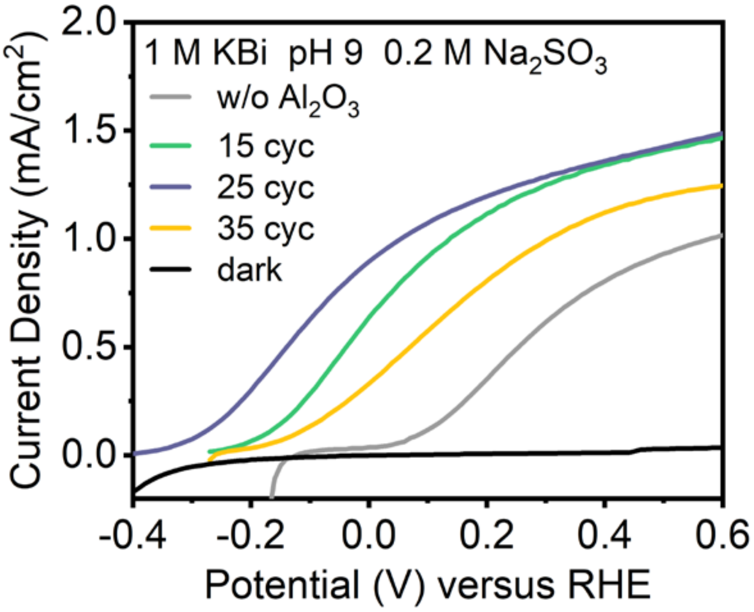

Figure S12. PEC SOR *J-V* curves of n-Si/Al_2O_3/ITO/$BiVO_4$ photoanode with different numbers of (TMA + H_2O) ALD cycles.

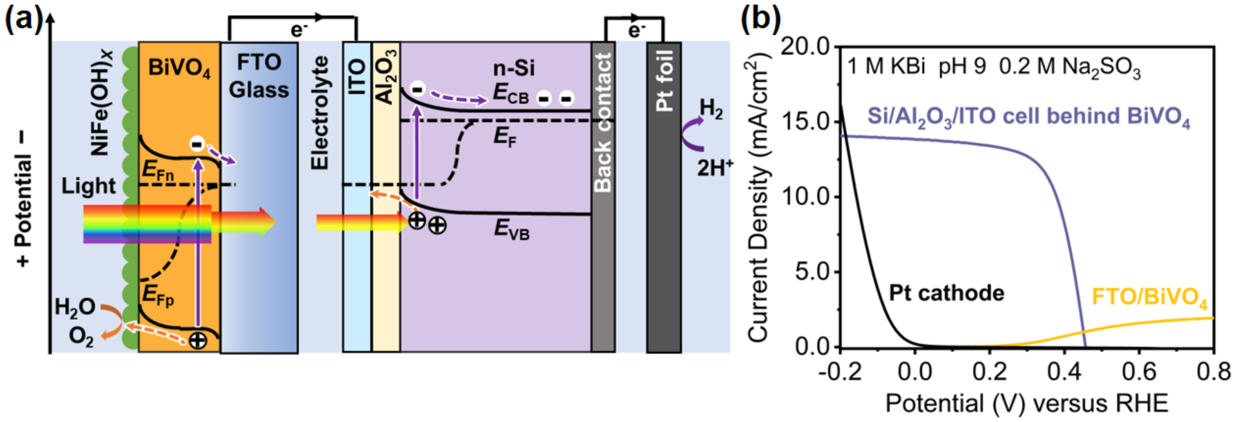

Figure S13. (a) Schematic energy band diagrams of the PV/PEC tandem water splitting cell consists of a $BiVO_4$ photoanode, a Si-based solar cell, and a Pt cathode. E_{CB} is the conduction band edge, E_{VB} is the valence band edge and E_F is the Fermi level. Layer thickness is not to scale, for clarity purposes. (b) PEC SOR *J*-*V* curves of the PV/PEC tandem cell under simulated AM 1.5 G illumination.

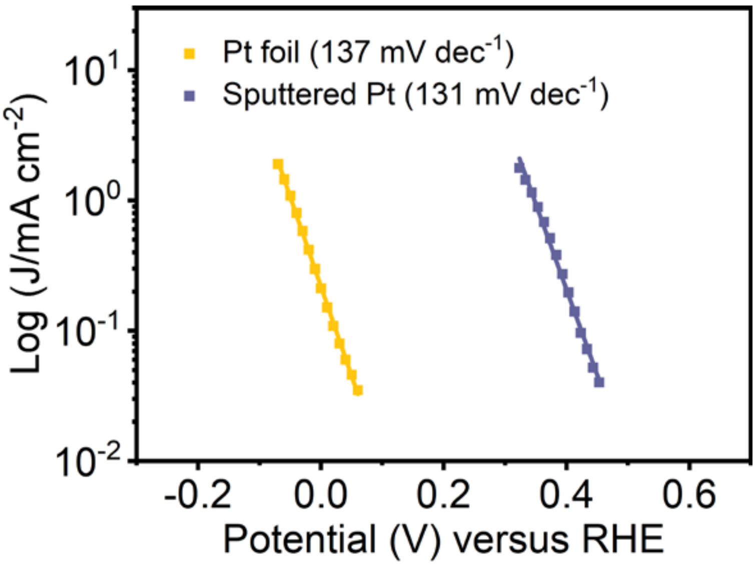

Figure S14. The corresponding Tafel plots of Pt foil and sputtered Pt layer on n-Si photocathode.

Table S1. Summary of recent representative reports on various PEC tandem cells for unbiased water splitting.

Photoanode	Photocathode	$J_{unbiased}$ (mA/cm^2)	Stability (h)	H_2 production (mL/cm^2)	Reference
$BiVO_4/NiFeO_x$	$SLG/Mo/CuIn_{0.5}Ga_{0.5}Se_2/CdS/TiO_2/Pt$	3	0.17	0.21	*Energy Environ Sci.* 2018[10]
$BiVO_4$/FeOOH/NiOOH	Si SHJ/TiO_2/Pt	1.5	0.25	0.16	*Energy Environ. Sci.* 2020[9]
np^+ b-Si/SnO_2/W-$BiVO_4$/Co-Pi	n^+-Si/Ti/CoP NPs	0.36	1	0.14	*Adv. Energy Mater.* 2017[7]
Mo:$BiVO_4$/FeOOH/NiOOH	pn^+-Si NWs/SiO_2+NiMo	1.71	1	0.72	*Nat. Energy* 2018[11]
$BiVO_4/CoO_x$	pn^+-Si/TiO_2/Pt	1.7	1.5	1.07	*J. Mater. Chem. A* 2020[12]
n-Si/$SiO_x/TiO_2/WO_3/BiVO_4$/Fe(Ni)OOH	Pt foil	0.2	2	0.14	*Adv. Energy Sustainability Res.* 2020[13]
$BiVO_4$/FeOOH/NiOOH	n-Si MIS/Ti/TiO_2/Pt	1.4	2	1.16	*Natl. Sci. Rev.* 2021[6]
$BiVO_4$/FeOOH/NiOOH	Si SHJ/TiO_2/Pt	2.8	10	11.93	*Angew.Chem.Int. Ed.* 2020[2]
$BiVO_4$/FeOOH/NiOOH	Si SHJ/TiO_2/Pt	3	10	12.61	*Adv. Funct. Mater.* 2020[14]
H,Mo:$BiVO_4/NiFeO_x$	$FTO/Au/Sb_2Se_3/CdS/TiO_2/Pt$	1.22	10	5.11	*Nat. Common.* 2020[15]
$BiVO_4/Co(OH)_2$	carbon-based PSC	3.7	10	15.68	*Electrochim Acta* 2020[16]
reduced Mo:$BiVO_4/NiFeO_x$	$FTO/Au/Cu_2O/Ga_2O_3/TiO_2/RuO_2$	2.5	12	12.27	*Nat. Catal.* 2018[17]
$BiVO_4$/CoPi	$CZTS/HfO_2/CdS/HfO_2/Pt$	2.6	20	21.81	*Energy Environ. Sci.* 2021[8]
$BiVO_4$/CoPi	$Cu_3BiS_3/CdS/TiO_2/Pt$	1.66	20	13.92	*Nat. Common.* 2021[18]
SnO_2/Mo:$BiVO_4$/NiFe	$Au/SnS/Ga_2O_3/TiO_2/Pt$	1.4	24	14.09	*Adv. Sci.* 2021[19]
Mo:$BiVO_4$/CTF-BTh/$NiFeO_x$	Cu_2O/CTF-BTh/MoS_x	3	120	150.93	*Adv. Mater.* 2021[20]
n-Si/Al_2O_3/ITO/$BiVO_4$/ NiFe$(OH)_x$	Pt foil	0.51	1045	223.43	This work

Table S2. Summary of recent representative reports on Si/metal oxides integrated photoanodes for PEC OER.

Integrated photoanode	Dark cathode	V_{onset} (V vs. RHE)	$J_{unbiased}$ (mA/cm^2)	Stability (h)	Electrolyte	Reference
n-Si NWs/TiO_2	Pt gauze	-0.26	0.07	-	1.0 M KOH	*Nano Lett.* 2009[21]
n-Si NWs/α-Fe_2O_3	Pt wire	0.6	0	-	1.0 M NaOH	*J. Am. Chem. Soc.* 2012[22]
n-Si NWs/α-Fe_2O_3	Pt foil	0.5	0	-	1.0 M NaOH	*Nanoscale* 2014[23]
n-Si/ITO/α-Fe_2O_3/IrO_x	Pt wire	0.82	0	-	1.0 M NaOH	*J.Catal.* 2020[24]
n-Si/WO_3	Pt wire	0.4	0	-	0.5 M H_2SO_4	*Adv. Energy Mater.* 2019[25]
np^+-Si MWs/ITO/WO_3	Pt foil	-0.04	0.017	0.17	1 M H_2SO_4	*Energy Environ. Sci.* 2014[26]
Si/TiO_2/$BiVO_4$	Pt foil	0.11	0	-	1 M Na_2SO_3 0.1 M KPi	*ACS Appl. Mater. Interfaces* 2015[27]
Si/TiO_2/$BiVO_4$/CoPi		0.09	0	-	0.1 M KPi	
n-Si/SnO_2/$BiVO_4$	Pt wire	-0.1	0.3	-	0.5 M KPi 1 M Na_2SO_3	*Energy Environ. Sci.* 2016[28]
n-Si/SnO_2/$BiVO_4$/Co-Pi		0.03	0	-	0.5 M KPi	
np^+ b-Si/SnO_2/W-$BiVO_4$	n^+-Si/Ti/ CoP NPs	-0.2	0.59	-	0.4 M Na_2SO_3 0.1 M KPi	*Adv. Energy Mater.* 2017[7]
np^+ b-Si/SnO_2/W-$BiVO_4$/Co-Pi		-0.16	0.36	1	0.1 M KPi	
n-Si/SiO_x/TiO_2/WO_3^{nano}/$BiVO_4$	Pt foil	-0.2	0.3	-	0.2 M Na_2SO_3 1 M KBi	*Adv. Energy Sustainability Res.* 2020[13]
n-Si/SiO_x/TiO_2/WO_3^{nano}/$BiVO_4$/Fe(Ni)OOH		-0.18	0.2	2	1 M KBi	
n-Si/Al_2O_3/ITO/$BiVO_4$	Pt foil	-0.35	1.29	-	0.2 M Na_2SO_3 1 M KBi	This work
n-Si/Al_2O_3/ITO/$BiVO_4$/$NiFe(OH)_x$		-0.17	0.51	1045	1 M KBi	

References

1. K. Sugiyama, H. Ishii, Y. Ouchi and K. Seki, *J. Appl. Phys.*, 2000, **87**, 295-298.
2. S. Feng, T. Wang, B. Liu, C. Hu, L. Li, Z. J. Zhao and J. Gong, *Angew. Chem. Int. Ed.*, 2020, **59**, 2044-2048.
3. X. X. Chang, T. Wang, P. Zhang, J. J. Zhang, A. Li and J. L. Gong, *J. Am. Chem. Soc.*, 2015, **137**, 8356-8359.
4. M. Li, T. Liu, Y. Yang, W. Qiu, C. Liang, Y. Tong and Y. Li, *ACS Energy Lett.*, 2019, **4**, 1983-1990.
5. Y. Hermans, S. Murcia-Lopez, A. Klein, R. van de Krol, T. Andreu, J. R. Morante, T. Toupance and W. Jaegermann, *Phys.Chem.Chem.Phys.*, 2019, **21**, 5086-5096.
6. S. Wang, T. Wang, B. Liu, H. Li, S. Feng and J. Gong, *Natl. Sci. Rev.*, 2021, **8**, nwaa293.
7. P. Chakthranont, T. R. Hellstern, J. M. McEnaney and T. F. Jaramillo, *Adv. Energy Mater.*, 2017, **7**, 1701515.
8. D. Huang, K. Wang, L. Li, K. Feng, N. An, S. Ikeda, Y. Kuang, Y. Ng and F. Jiang, *Energy Environ. Sci.*, 2021, **14**, 1480-1489.
9. B. Liu, S. Feng, L. Yang, C. Li, Z. Luo, T. Wang and J. Gong, *Energy Environ. Sci.*, 2020, **13**, 221-228.
10. H. Kobayashi, N. Sato, M. Orita, Y. Kuang, H. Kaneko, T. Minegishi, T. Yamada and K. Domen, *Energy Environ. Sci.*, 2018, **11**, 3003-3009.
11. W. Vijselaar, P. Westerik, J. Veerbeek, R. M. Tiggelaar, E. Berenschot, N. R. Tas, H. Gardeniers and J. Huskens, *Nat. Energy*, 2018, **3**, 185-192.
12. H. Li, B. Liu, S. Feng, H. Li, T. Wang and J. Gong, *J. Mater. Chem. A*, 2020, **8**, 224-230.
13. I. Y. Ahmet, S. Berglund, A. Chemseddine, P. Bogdanoff, R. F. Präg, F. F. Abdi and R. van de Krol, *Adv. Energy Sustainability Res.*, 2020, **1**, 2000037.
14. B. Liu, S. Wang, S. Feng, H. Li, L. Yang, T. Wang and J. Gong, *Adv. Funct. Mater.*, 2020, **31**, 2007222.
15. W. Yang, J. H. Kim, O. S. Hutter, L. J. Phillips, J. Tan, J. Park, H. Lee, J. D. Major, J. S. Lee and J. Moon, *Nat. Commun.*, 2020, **11**, 861.
16. X. Li, M. Jia, Y. Lu, N. Li, Y.-Z. Zheng, X. Tao and M. Huang, *Electrochim. Acta*, 2020, **330**, 135183.
17. L. Pan, J. H. Kim, M. T. Mayer, M.-K. Son, A. Ummadisingu, J. S. Lee, A. Hagfeldt, J. Luo and M. Grätzel, *Nat Catal*, 2018, **1**, 412-420.
18. D. Huang, L. Li, K. Wang, Y. Li, K. Feng and F. Jiang, *Nat. Commun.*, 2021, **12**, 3795.
19. H. Lee, J. W. Yang, J. Tan, J. Park, S. G. Shim, Y. S. Park, J. Yun, K. Kim, H. W. Jang and J. Moon, *Adv. Sci.*, 2021, **8**, 2102458.
20. Y. Zhang, H. Lv, Z. Zhang, L. Wang, X. Wu and H. Xu, *Adv. Mater.*, 2021, **33**, 2008264.
21. Y. J. Hwang, A. Boukai and P. Yang, *Nano Lett.*, 2009, **9**, 410-415.
22. M. T. Mayer, C. Du and D. Wang, *J. Am. Chem. Soc.*, 2012, **134**, 12406-12409.
23. X. Qi, G. She, X. Huang, T. Zhang, H. Wang, L. Mu and W. Shi, *Nanoscale*, 2014, **6**, 3182-3189.
24. D. Feng, J. Qu, R. Zhang, X. Sun, L. Zheng, H. Liu, X. Zhang, Z. Lu, F. Lu, W. Wang, H. Dong, Y. Cheng, H. Liu and R. Zheng, *J. Catal.*, 2020, **381**, 501-507.
25. Y. Zhao, G. Brocks, H. Genuit, R. Lavrijsen, M. A. Verheijen and A. Bieberle-Hütter, *Adv. Energy Mater.*, 2019, **9**, 1900940.
26. M. R. Shaner, K. T. Fountaine, S. Ardo, R. H. Coridan, H. A. Atwater and N. S. Lewis, *Energy Environ. Sci.*, 2014, **7**, 779-790.
27. H. Jung, S. Y. Chae, C. Shin, B. K. Min, O. S. Joo and Y. J. Hwang, *ACS Appl. Mater. Interfaces*, 2015, **7**, 5788-5796.
28. L. Zhang, X. Ye, M. Boloor, A. Poletayev, N. A. Melosh and W. C. Chueh, *Energy Environ. Sci.*, 2016, **9**, 2044-2052.
